# The Potential for Cellular Therapy Combined with Growth Factors in Spinal Cord Injury

**DOI:** 10.1155/2012/826754

**Published:** 2012-10-03

**Authors:** Jack Rosner, Pablo Avalos, Frank Acosta, John Liu, Doniel Drazin

**Affiliations:** ^1^Department of Neurosurgery, Cedars-Sinai Medical Center, 8363 West 3rd Street Ste 800E, Los Angeles, CA 90048, USA; ^2^Regenerative Medicine Institute, Cedars-Sinai Medical Center, Los Angeles, CA 90048, USA

## Abstract

Any traumatic spinal cord injury (SCI) may cause symptoms ranging from pain to complete loss of motor and sensory functions below the level of the injury. Currently, there are over 2 million SCI patients worldwide. The cost of their necessary continuing care creates a burden for the patient, their families, and society. Presently, few SCI treatments are available and none have facilitated neural regeneration and/or significant functional improvement. Research is being conducted in the following areas: pathophysiology, cellular therapies (Schwann cells, embryonic stem cells, induced pluripotent stem cells, mesenchymal stem cells, olfactory ensheathing cells), growth factors (BDNF), inhibitory molecules (NG2, myelin protein), and combination therapies (cell grafts and neurotrophins, cotransplantation). Results are often limited because of the inhibitory environment created following the injury and the limited regenerative potential of the central nervous system. Therapies that show promise in small animal models may not transfer to nonhuman primates and humans. None of the research has resulted in remarkable improvement, but many areas show promise. Studies have suggested that a combination of therapies may enhance results and may be more effective than a single therapy. This paper reviews and discusses the most promising new SCI research including combination therapies.

## 1. Introduction

Spinal cord injury (SCI) is defined as any traumatic injury to the spinal cord. Depending on the level of the spine at which it occurs and the severity of the insult, SCI may cause symptoms ranging from pain to complete loss of motor and sensory functions below the level of the injury. In the United States, incidence of SCI is approximately 40 cases per million individuals per year, resulting in about 12,000 new cases per year [1]. It is important to note that this figure does not take into account injuries that result in death prior to hospital admission and thus underestimates total incidence of SCI. Nationally and internationally, the leading cause of SCI is traffic accidents, which account for half of all injuries. Recreational activities, falls, violence, and work-related accidents account for the majority of the remainder of SCI [2].

The majority of SCIs occur in young individuals with the mean age at the time of injury at 35.3 years [2]. This young onset of lifelong debilitation results in particularly high personal and economic costs which are very high. Over the past three decades, there has been a substantial decline in mortality during the first 2 years following injury [3] and life expectancy following SCI now approaches that of the population at large [4]. Currently, there are over 2 million living survivors of SCI worldwide [5], many of which require some form of continuing care. Additionally, rehospitalization following SCI is common, with the leading causes being genitourinary, respiratory, and skin infections [6]. As of the late 1990s, the average lifetime cost of treating SCI was estimated at between $500,000 and $2 million per patient with total costs exceeding $7 billion per year in the United States alone [7].

Although there are few therapies for the underlying injury, traditional management of SCI includes prevention of reinjury, treatment of secondary complications, and provision of providing rehabilitative therapy [7, 8]. Corticosteroid treatment has been investigated as a means of controlling inflammation after SCI and thereby lessening the severity of the initial injury. Methylprednisolone administered shortly after SCI may provide a modest improvement in sensory and motor function below the level of the injury at followup [9, 10], although a number of investigators have questioned the efficacy of this intervention [11–13] and it has been suggested that further research is needed to definitively evaluate the role of corticosteroids in SCI management [14]. 

In recent years, a better understanding of the mechanisms underlying SCI and the introduction of tissue engineering, stem cell, growth factor, anti-inhibitory and combination therapies have led to a number of new investigatory avenues for SCI treatment. Numerous studies have suggested that, going forward, the most effective treatment for SCI may involve a combination of novel therapies [15, 16]. In this paper, we review the most promising new treatments for SCI and suggest further investigations using combination therapies.

## 2. Pathophysiology

SCI pathophysiology can be divided into two phases. The initial injury constitutes the primary phase. During this phase, a precipitating event results in a mechanical force on the spine that causes damage to the spinal cord, resulting in the disruption and death of vasculature and neural elements. Depending on the etiology of the injury, this force may cause compression, contusion, laceration, or transection of the cord, although even in patients suffering complete paralysis, full transections are rare. Most commonly, the primary injury involves an initial contusion followed by persisting compression [2]. 

While the primary injury may cause observable damage to the cord, there are often no histological abnormalities immediately following SCI [17]. This suggests the importance of the secondary phase in SCI pathology. The secondary phase begins hours after the initial insult and is marked by a number of cellular and molecular changes in and around the injured area. Ongoing investigation is continuing to illuminate the underlying causes of these changes and they will not be reviewed in detail here. Some mechanisms investigated include vascular abnormalities [18], free radicals causing oxidative stress [19], and glutamate excitotoxicity [20].

Observed effects of the secondary phase include apoptosis, Wallerian degeneration, and the formation of a glial scar. Active apoptosis of oligodendrocytes resulting in demyelination and further degeneration of white and gray matter up to 13 mm from the lesion center has been observed to peak at 8 days and continue until at least three weeks following injury [21, 22]. Demyelination is particularly disruptive to the ability of spinal cord neurons to transmit electrical impulses [23] and represents a significant therapeutic target.

Wallerian degeneration is another secondary process following SCI. Wallerian degeneration refers to the breakdown and demyelination of axons distal to an injury. This injury could be to a cell body or to a portion of the axon proximal to a cell body. In SCI, Wallerian degeneration is seen both above and below the level of the injury (above in dorsal columns, below in corticospinal tracts). It is not observed by MRI until 7 weeks following injury [24] and likely continues for years thereafter [25]. 

The secondary phase culminates in the formation of a glial “scar.” A universal CNS injury response, the presence of a glial scar, has a number of implications for treatment of SCI, and thus it is helpful to understand its composition and effects. Several days after SCI, astrocytes at the edges of the lesion begin to hypertrophy and undergo a number of morphological and molecular changes. New astrocytes also appear, although their lineage is unclear [26]. One distinctive molecular change is the increased expression of glial fibrillary acidic protein (GFAP) [27], a cytoskeletal component necessary for cell growth. Over the next several weeks, these astrocytes extend overlapping processes and form a dense network of gap and tight junctions that constitute the scar [28]. Although composed primarily of astrocytes, the scar also contains oligodendrocytes, oligodendrocyte precursors, and microglia, among other cell types.

The glial scar has both beneficial and detrimental effects. By isolating the site of injury, the glial scar is instrumental in restoring the blood brain barrier, and there is also evidence that it removes excitotoxic glutamate, helps protect neural cells from oxidative stress, and stabilizes extracellular ion balance, thereby reducing the seizure threshold [26, 29]. Unfortunately, the scar also presents an impediment to axonal regrowth. In addition to acting as a physical barrier, the glial scar secretes a number of molecules that inhibit axonal sprouting, including tenascin, Semaphorin 3, and keratin and chondroitin sulfate proteoglycans (KSPGs and CSPGs) such as NG2 [30, 31]. 

Because the secondary response to SCI unfolds over an extended period following injury and therefore may theoretically be subject to clinical management, novel treatments for SCI have focused primarily on regulating the cellular and molecular changes that mark this phase.

## 3. Biomaterials

Tissue engineering is one strategy being explored to treat SCI. At the most basic level, polymer-based biomaterials may bridge physical gaps at the injury site, providing a substrate for neural regeneration. Features of attractive biomaterials are biocompatibility, biodegradability, and a high binding capacity. Broadly, two types of biomaterials have been tested for SCI. The first are compounds like fibrin and collagen, which are viscous and boast the ability to gel in situ, facilitating less invasive administration and conforming to the injury site [32]. The second group of compounds are premolded and designed to provide a structured scaffolding to support neural element growth. Examples of these are poly(lactic-co-glycolic acid) (PLGA) and agarose.

A number of biomaterials have been shown to promote neural growth and delay the accumulation of reactive astrocytes when implanted at a lesion site [32–34]. In addition to these histological features, some studies have also observed functional recovery in small animals treated with biomaterials alone [34]. However, there is widespread consensus that biomaterials are most promising when used as vehicles for neurotrophin delivery and scaffolding for glial cell grafts, strategies that will be discussed as combinational therapies.

## 4. Cellular Therapies

There are three main goals of cellular therapy for SCI. Firstly, grafted cells may directly replace neural elements such as neurons or oligodendrocytes lost through primary or secondary mechanisms. Secondly, cell grafts help to fill in lesions and provide a scaffolding to support endogenous neural element growth. Lastly, grafted cells can create an environment more conducive to neuroprotection, regrowth, and remyelination. A number of cell types have been investigated for transplantation after SCI. Among these are Schwann cells (SCs) [32–37], embryonic stem cells (ESCs) [38, 39], mesenchymal stem cells (MSCs) [40–42], and olfactory ensheathing cells (OECs) [15, 36, 43–45]. 

### 4.1. Schwann Cells

Schwann cells normally serve to myelinate the peripheral nervous system and have long been known to be capable of remyelinating and facilitating axonal regeneration of injured spinal cord neurons [46, 47]. Demyelination significantly impairs axonal conduction [23] and thus represents a major therapeutic target following SCI. Studies in rats have found remyelination of spinal axons following SC transplantation in several models of SCI [32–37], and axons regenerated within SC grafts are capable of conducting action potentials [48]. Modest functional improvements were reported in these same studies [32, 36]. Additionally, SCs seem to provide effective scaffolding for both sensory and motor axonal regrowth following complete transection and also assist in remyelination in this context [37]. 

### 4.2. Embryonic Stem Cells

Embryonic stem cells (ESCs) are pluripotent cells that can be harvested from the blastocyst inner cell mass. Undifferentiated ESCs form teratomas when grafted in vivo [49, 50] and therefore must be induced towards a neural lineage prior to grafting. Neural differentiated ESCs transplanted into injured rat spinal cords have been found to differentiate into astrocytes, oligodendrocytes, and neurons [39] and have been associated with moderate locomotor recovery [38, 39]. ESC-derived oligodendrocytes have been shown to assist in remyelination following chemically induced demyelination [51] and SCI [38] in the rat spinal cord. 

### 4.3. Induced Pluripotent Stem Cells

Induced pluripotent stem cells (IPS) are becoming a widespread alternative to embryonic stem cells. IPS cells are adult somatic cells that have been reprogrammed by a combination of factors to become pluripotent and are able to generate any cell type when differentiated. IPS lines can be derived from the patient's own somatic cells. Therefore, although not well-known, IPS cells may overcome the immunological concerns associated with cellular therapy. Another advantage to the use of IPS cells is that they can circumvent the ethical controversies that are associated with embryonic stem cells. As with ESCs, undifferentiated IPS cells form teratomas when grafted in vivo [52]. IPS-derived astrocytes have been transplanted into a rat SCI model. Although the cells were not rejected and engrafted successfully (as shown by the extension of processes), there was no evidence of functional recovery. Sensitivity to mechanical stimuli, however, was increased [52]. The lack of functional recovery may be explained by the absence of other cells types. Human and mouse IPS-derived neural progenitor cells were transplanted into a mouse SCI model and were shown to differentiate into electrophysiologically functional neurons, astrocytes and oligodendrocytes [53]. Synapse of the transplanted, neurons and host neurons was accomplished along with increased angiogenesis, remyelination, axonal regeneration, and recovery of locomotion function [53, 54]. 

### 4.4. Mesenchymal Stem Cells

Mesenchymal stem cells (MSCs), also referred to as bone marrow stromal cells (BMSCs), represent an attractive stem cell source because they are relatively easy to harvest, display persistent engraftment, and avoid the ethical issues associated with the harvesting of embryonic stem cells [19, 55, 56]. Additionally, MSCs have been shown in vitro to be capable of differentiating into cells exhibiting neuron morphology and several neuron-specific proteins [15], although whether these cells are able to generate action potentials is questionable [41]. It is also not clear if such differentiation can occur in vivo [42]. In animal models, MSC transplantation following SCI has been observed to promote axonal regeneration [37–39] and is associated with functional recovery [40, 41]. Human MSCs (hMSCs) have also been investigated in small animal models in anticipation of future translational research and were found to be well tolerated and to promote limited functional recovery [57, 58]. 

The mechanism of MSC-mediated regeneration is unclear. MSCs have been observed to promote the differentiation of neural stem cells (NSCs) in vitro [42], suggesting that a similar action may occur in vivo. Even the presence of unmodified MSCs in CNS tissue increases concentrations of neurotrophins including BDNF, NGF, and NT-3 in the graft site [15, 57]. Thus, it is difficult to assess MSC efficacy separately from that of neurotrophins, and it is likely that improvements in neural regeneration observed with MSCs are due at least in part to increased neurotrophin concentration.

### 4.5. Olfactory Ensheathing Cells

Olfactory ensheathing cells (OECs), also known as olfactory ensheathing glia (OEG), are found both centrally and peripherally along the olfactory nerve. Their candidacy for SCI repair rests largely on their observed ability to facilitate continuous neurogenesis in the inhibitory environment of the mature CNS [59]. Several trials using OEC grafts to treat SCI in rats have yielded encouraging results, with observed corticospinal axon regeneration, remyelination, and functional locomotor improvements [15, 36, 43–45]. More recently, a number of foreign trials using OECs for SCI have been conducted in humans, the largest of these involving over 300 patients in China [60], although there is concern about the methodological standards of these trials; many are poorly controlled, have not been subject to independent analysis, or use unscientific selection criteria or outcome measures [61]. Thus, few definite conclusions can be drawn from this work.

More recent studies have cast doubt on whether the improvements observed in animal trials of OECs are direct or indirect effects of OEC engraftment. Direct remyelination was proposed as a major mechanism for the early effectiveness of OECs [43, 45, 62]; however, it has been found that OEC cultures likely contain SCs and therefore that those SCs may be the source of the observed myelination [46]. It is further hypothesized that OECs may help recruit additional endogenous SCs, further facilitating myelination, although the mechanism of this recruitment is not known [63]. Thus, the primary value of OEC transplantation may actually lie in their recruitment of SCs.

## 5. Growth Factors

Growth factor therapy has shown significant promise in the treatment of a number of CNS conditions including Alzheimer disease [64] and ischemic stroke [65]. In SCI treatment, the primary role of growth factors is to first promote neuronal survival, and, later, axonal regrowth. 

A number of growth factors have been investigated to treat SCI. The most researched are brain-derived neurotrophic factor (BDNF) [66–72], nerve growth factor (NGF) [69, 73], neurotrophin-3 (NT-3) [67, 69, 70, 74], glial cell-derived neurotrophic factor (GDNF) [75], and basic fibroblast growth factor (bFGF) [73, 76, 77]. While numerous trials of growth factors for SCI have been performed in vitro and using small and, more recently, large animal models, many questions remain regarding the efficacy of these treatments and the optimal conditions for their administration.

BDNF is one of the more studied and promising growth factors for SCI therapy. In small animal models, BDNF appears to increase motor functioning shortly after injury; however, the statistical significance of this effect disappears at later followups [66, 69]. These results point to the neuroprotective effects of BDNF immediately following injury. One possible mechanism of this neuroprotection is the recruitment of oligodendrocytes and the ensuing increase in myelination of damaged and growing axons [67], and the drop-off in improvement over control at later followups suggests that BDNF alone is not sufficient to spur axonal regrowth. Other studies, however, have demonstrated BDNF-induced axonal regrowth in the presence of a fibroblast graft, indicating that BDNF is capable of effecting such regeneration in a suitable environment [67, 72]. Data on other growth factors is less robust but still promising (Table 1).

Interestingly, different axon systems respond to growth factors differently. Corticospinal axons—those originating in the cerebral cortex and carrying voluntary muscle movements—have in particular been generally unresponsive to therapy using a number of growth factors in both small animal and primate models [70, 75]. BDNF does appear to provide a neuroprotective effect [70] and studies have found some corticospinal axon regrowth accompanied by mild functional improvement in response to NT-3 in rats [78, 79].

One consideration with the use of all growth factors is the establishment of an appropriate concentration gradient. A number of studies have observed that, in the presence of neurotrophin-secreting grafts, axons will regenerate into the graft but not beyond it [67, 75]. This might be explained by the finding that ascending neurotrophic concentration gradients guide neurite outgrowth [80], meaning that axonal growth cones may become “trapped” in neurotrophin-secreting grafts where the concentration of growth factor is higher than in the surrounding endogenous tissue. One possible solution to this problem is the use of biomaterial scaffolding containing a preexisting, immobilized concentration gradient of neurotrophins to guide neurons through the lesion, a strategy that has shown promise in vitro [81, 82].

Beyond neuroprotection and the stimulation of axonal growth immediately following injury, growth factor therapy may also help overcome some of the challenges presented by the mature glial scar's secretion of inhibitory factors. In the presence of NT-3, Lu et al. [74] found that axons penetrated a mature glial scar 3 months following injury and extended into an MSC-grafted lesion cavity. The authors hypothesized that regulation of neurite outgrowth may depend on the balance between inhibitors and other molecules including neurotrophins, an idea which has also been suggested by other studies examining neural growth in the presence of CSPGs [31]. Kwon et al. [72] also found atrophied rubrospinal neurons capable of regenerating into peripheral nerve grafted lesions following administration of BDNF one year following injury. Tobias et al. [71] found a similar effect with BDNF and NT-3 in a fibroblast-grafted lesion 6 weeks following injury, although the authors note that this regeneration did not compare favorably to similar treatments administered acutely. 

Taken together, these studies demonstrate that growth factors function neuroprotectively immediately following injury, as well as playing an important role in neural regeneration in the mature injury site. Functional improvement is also associated with use of growth factors in some animal models, although these findings are often borderline significant and evaluated using varying metrics and therefore must be received cautiously.

## 6. Inhibitory Molecules

The presence of axonal outgrowth inhibitors also seems to play a major role in the lack of regeneration after SCI. A key obstacle in finding a successful therapy for SCI has been the presence of these inhibitory molecules following injury. The most notable molecules that have been described are NG2 [83], myelin-associated glycoprotein (MAG) [84–86], NogoA [87–90], and OMgp [91, 92]. Optimal therapy for SCI might require the inclusion, at least in part, of inhibition of these molecules. 

### 6.1. NG2

NG2 is an inhibitory molecule that belongs to the chondroitin sulfate proteoglycan family and is expressed after CNS injuries. It is mostly expressed on the surfaces of oligodendrocytes and macrophages and has been associated with inhibition of neuronal outgrowth after SCI. Jones et al. showed that NG2 expression is upregulated within 24 hours of injury and it increases over the following two weeks after injury [93]. Studies using rat cerebellar granule neurons show that neurite growth is inhibited by the presence of NG2 even on laminin-coated surfaces by preventing neuronal cell attachment and elongation of axons [83]. The inhibitory dominance created by NG2 was further shown in a rat model of SCI where the transected CST axons were found surrounding an area of high NG2 expression [93]. Therefore, therapy focused on reducing the levels of NG2 in response to spinal cord injury may lead to improved axonal regeneration. 

### 6.2. Myelin Proteins

Nogo-A, oligodendrocyte myelin glycoprotein (OMgp), and myelin-associated glycoprotein (MAG) are expressed by oligodendrocytes after injury to the adult CNS and are known as myelin-associated proteins (MAPs). These myelin-associated proteins are culprits that lead to inhibition of axonal regeneration by blocking axonal growth after injury [94–96]. All three MAPs bind to the Nogo-66 receptor (NgR), a receptor that is anchored to the membrane via a glycosylphosphatidylinositol (GPI) linkage [87–91, 97]. It appears that the three proteins compete with each other for binding with the receptor [94, 98]. Sharing of the receptor may explain why therapy targeted at a specific protein only has subtle effects on the axonal regeneration inhibition. However, since NgR is a point of convergence for the three different MAPs, it makes it an appealing target for therapy. There have been several therapies that have been studied by different groups showing efficacy in vivo.

GrandPre et al. [89] proved that using an NgR antagonist prevents inhibition by all three MAPs. Furthermore, administration of the soluble function-blocking NgR domain in a rat SCI model increases axonal sprouting leading to improved electrical spinal cord conduction and locomotion [97]. Infusion of the monoclonal antibody against Nogo, IN-1 into the lesion site of a rat's spinal cord improves axonal outgrowth and functional recovery following SCI suggesting increased plastic and regenerative capabilities of the CNS [85, 97]. Nevertheless, the improvements were subtle most likely due to the presence of MAG and OMgp binding to the NgR [99, 100]. MAG and Nogo-A knockout mice have been studied and have revealed that the inhibitory effects of MAG and Nogo-A, respectively, were decreased leading to improved axonal regeneration [101–103]. In the case of the Nogo-A knockout mouse, the decreased inhibitory effects were very similar to those seen with Nogo-A specific antibodies. Rho pathway inhibitors and cAMP elevation are some other therapies that have shown some efficacy in vivo [104–107]. Supplementary evidence to the benefits of anti-inhibitory therapy has surfaced by the addition of anti-MAG antibodies to cultures in vitro leading to a reversal of outgrowth inhibition by about 50% [85].

The benefits seen from using anti-inhibitory therapies are vast, in particular therapies that inhibit all three proteins by blocking the NgR. Administration of anti-inhibitory therapies alone or in combination with growth factors, cellular therapy or both could be the needed solution to recovery from spinal cord injury. 

## 7. Combination Therapies

Recently, there has been increasing recognition that the most promising treatment for SCI may combine a number of novel therapies that, while modestly effective individually, have an enhanced effect when used together. So-called combination therapies aim to create a neuroprotective environment, foster regeneration, and counter inhibitory factors released after CNS injury. All of these are likely required for robust regeneration following SCI. These therapies may involve neurotrophins and cell grafts [15, 108–110], cotransplantation of different grafts [111–113], or anti-inhibitory therapies. 

### 7.1. Cell Grafts and Neurotrophins

Various cell grafts transduced to secrete growth factors or transplanted with exogenous growth factor administration have also shown increased efficacy versus cell grafts alone. Weidner et al. [109] investigated SCs transduced to secrete NGF in a rat model and found significantly increased neuronal growth into grafts of secreting versus nonsecreting SCs. Xu et al. [110] found that a combination of BDNF and NT-3 administered continuously following SC grafting improved neural ingrowth and myelination as compared to SC or neurotrophin-only controls. BDNF-secreting MSCs have shown promising results in a rat model. More robust neural growth was observed into grafts of MSCs induced to secrete BDNF versus control MSCs, although how this effect compares to treatment with only BDNF was not part of the study design [114]. Another study found that SCs transduced to secrete D15A, a neurotrophin that mimics NT-3 and BDNF, engendered more robust regeneration (greater graft volume, increased myelination, greater axon length) when compared to unmodified SC grafts [108]. 

### 7.2. Cotransplantation

Grafts employing combinations of cell types also appear advantageous over single-cell-type grafts. Pearse et al. [112] investigated a combination of SCs and OECs and reported significantly greater functional improvement in SC and OEG graft subjects versus controls and only SC or OEC graft subjects. Deng et al. [111] found that cotransplantation of MSCs with OECs in a rat model resulted in greater functional improvement versus either cell type alone. The authors suggest one possible reason for this was that the OECs had a positive effect on MSC survival and neural differentiation. Zeng et al. [113] have proposed a similar mechanism to explain the finding that SCs transplanted with neural stem cells lead to increased neural stem cell growth and differentiation.

### 7.3. Biomaterials with Neurotrophins and Cell Grafts

Frequently, biomaterial scaffolding has been used in conjunction with other regenerative and anti-inhibitory treatments, such as neurotrophin administration [115–119] and glial cell grafts [120, 121].

Two significant problems with the use of growth factors to treat SCI have been the maintenance of adequate concentrations following administration and the establishment of an appropriate concentration gradient for directed neural outgrowth. Scaffolding strategies offer promising solutions to these problems. Through sustained release mechanisms such as heparin-based delivery systems (HBDSs), several groups have had success establishing the diffusion- and cell-mediated release of neurotrophins. Taylor et al. [117, 118] tested an NT-3 secreting fibrin gel using a HBDS administrated immediately after injury and found significantly increased neuronal sprouting compared to non-HBDS NT-3 fibrin and fibrin only controls. Similarly, rats treated with collagen-bound BDNF (facilitating sustained delivery) displayed significant nerve fiber growth and functional improvement over use of the scaffold alone [115]. 

Other studies have incorporated neurotrophins into rigid scaffolds designed to guide axonal growth. When BDNF was incorporated into the walls and lumen of a freeze-dried agarose scaffold and implanted following SCI, axons were observed to grow through individual small-diameter channels, suggesting the ability of properly organized biomaterials to support a tract-like axonal layout similar to that within the uninjured spinal cord [116]. Beyond direct implantation in the scaffold, other methods of neurotrophin delivery are being explored. Tuinstra et al. [119] investigated the use of lentiviral vectors encoding NT-3 and BDNF delivered through multichannel PLGA scaffolds in a rat hemisection model. Compared to empty scaffolds, scaffolds with vectors had significantly more axons per channel with enhanced myelination. This is consistent with the observed effects of NT-3 and BDNF, suggesting the efficacy of this unique delivery method.

The injured spinal cord appears responsive to such biomaterial-neurotrophin therapies even when there is a significant delay to treatment. Johnson et al. [122] found that rats treated with an NT-3 releasing fibrin scaffold 2 weeks after the initial insult showed an increase in neural fiber density and a decrease in astrocyte accumulation, although this study did not investigate functional recovery.

Biomaterials have also shown promise when used to enhance cellular therapies. Hemisected rats injected with a fibrin matrix containing BMSCs had significantly greater functional recovery at up to 4 weeks (endpoint) following transplantation than those treated with fibrin or BMSCs alone. Histological analysis further revealed enhanced survival and migration of fibrin-bound BMSCs versus those injected alone [120]. Highly organized scaffold designs have also shown promise when used with cell grafts. Teng et al. [121] investigated a PLGA scaffold seeded with NSCs in a rat hemisection model. The scaffold contained a core designed to facilitate axonal growth and an outer portion designed to allow fluid transport while inhibiting scar tissue growth. The most significant axonal growth and functional improvement were noted in the seeded scaffold group, although the presence of an empty scaffold also facilitated some improvement over control. The authors hypothesize that the major contribution of the NSCs was to provide trophic support to existing neural elements rather than replacing them directly, as most NSCs remained undifferentiated.

**Figure 1:**
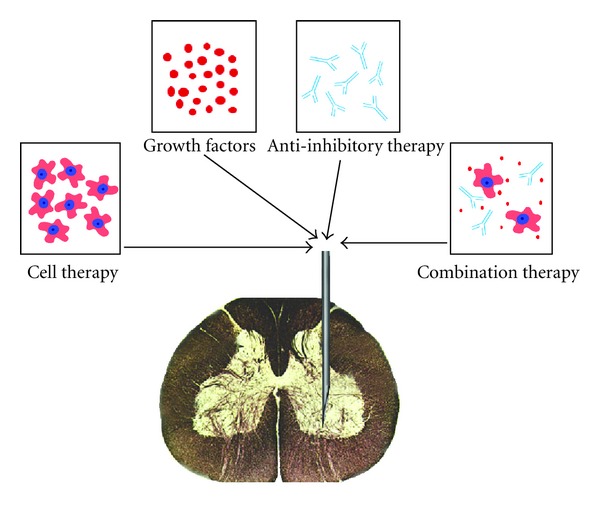
The individual and combination therapies currently being studied which show great promise.

In short, studies to date indicate that combination therapies pose no additional dangers over their constituent interventions and seem to be, theoretically, more efficacious (Figure 1). Although, more studies are required, the authors believe that the best combination therapy could be a biomatrix scaffold, with sustained release of neurotrophic factors (BDNF and NT-3), which is seeded with a cellular graft. The cell type choice is still undetermined due to the lack of studies determining a clear benefit from one cellular type to another.

## 8. Conclusion

Facilitating significant neural regeneration and ensuing functional improvement following spinal cord injury remains a challenging goal. Because of the inhibitory environment created following CNS injury including SCI and the intrinsically limited regenerative potential of the CNS (e.g., the insufficient expression of regeneration associated genes such as those coding for cytoskeletal proteins) [123], strategies to regenerate neurons and other neural elements face a twofold challenge.

There is also still a great deal of uncertainty regarding how therapies investigated thus far primarily in small animal models will transfer to nonhuman primates and, eventually, humans. While some preliminary trials have been conducted, they are either poorly reported or have not lead to remarkable improvement. Nevertheless, there is certainly reason to be optimistic regarding the possibility of combining new therapeutic approaches for SCI.

## Figures and Tables

**Table 1:** A review of the published studies on growth factors and spinal cord injury.

Reference	Model (animal/injury/level)	Factor	*N*	Graft	Histological effect	Functional effect
Grill et al., [78]	Rat/dorsal hemisection and dorsal column lesion/T7	NT3	21	Fibroblast	Corticospinal axon growth	Locomotor score improvement

Houweling et al., [66]	Rat/partial transection/T9	BDNF	9	NA	None	Locomotor score improvement within 1 week of injury; none at later f/u

McTigue et al., [67]	Rat/contusion/T8	NT3	6	Fibroblast	Cell growth into graft, remyelination	NA
		BDNF	12	Fibroblast	Cell growth into graft, remyelination	NA
		bFGF	5	Fibroblast	None	NA
		NGF	5	Fibroblast	None	NA

Lee et al., [73]	Rat/contusion/T10	bFGF	5	NA	Reduction in necrosis	NA
		NGF	5	NA	None	NA

Liu et al., [68]	Rat/partial hemisection/C3	BDNF	72	Fibroblast	Rubrospinal axon regeneration	Partial recovery of forelimb function

Rabchevsky et al., [76]	Rat/contusion/T10	bFGF	18	NA	Reduction in necrosis	Partial recovery of motor function at all f/u (up to 6 weeks)

Namiki et al., [69]	Rat/compression/T3	BDNF	6	NA	None	Higher inclined plane score 1 week following injury; none at later f/u
		NGF	6	NA	None	None
		NT3	6	NA	None	None

Rabchevsky et al., [77]	Rat/contusion/T10	bFGF	16	NA	None	Partial recovery of motor function at all f/u (up to 6 weeks)

Tuszynski et al., 2002	Rat/dorsal hemisection /T7	NT3	66	Fibroblast	Corticospinal axon growth	Locomotor score improvement

Blesch and Tuszynski, [4]	Rat/complete transection and dorsal hemisection/T7	GDNF	44	Fibroblast	Cell growth into graft, remyelination	None at 4 weeks (first f/u) and beyond.

Brock et al., [70]	Primate/lateral hemisection/C7	BDNF & NT3	7	Fibroblast	Neural growth into graft, neuroprotection of corticospinal neurons	NA
